# Antimicrobial, Anti-Inflammatory, General Toxicity and Antioxidant Activities of Ultrasound-Assisted Extracts from *Prunella vulgaris* L.

**DOI:** 10.3390/ph19101646

**Published:** 2026-10-19

**Authors:** Mussa E. Zholdasbayev, Roza B. Seidakhmetova, Gayane A. Atazhanova, Svetlana A. Ivasenko, Saule B. Akhmetova, Ardak O. Sapiyeva, Tobylzhan G. Salikov, Nazigul S. Remetova, Yana K. Levaya, Dmitry A. Kudlay, Elena A. Smolyarchuk, Aleksandr V. Samorodov

**Affiliations:** 1 School of Pharmacy, NCJSC Karaganda Medical University, Karaganda 100012, Kazakhstan; rozabat@mail.ru (R.B.S.); g-atazhanova@mail.ru (G.A.A.); ivasenko-74@mail.ru (S.A.I.); akhmetova_sb@mail.ru (S.B.A.); tobylzhan.95@gmail.com (T.G.S.); remetnaziko@mail.ru (N.S.R.); yaninka_25@mail.ru (Y.K.L.); 2 Department of Pharmacology, A.P. Nelyubin Institute of Pharmacy, Sechenov First Moscow State Medical University ( Sechenov University), Moscow 119991, Russia; d624254@gmail.com (D.A.K.); smolyarchuk@mail.ru (E.A.S.); avsamorodov@gmail.com (A.V.S.); 3 Department of General and Biological Chemistry, NCJSC Sharmanov National Medical University, Astana 010000, Kazakhstan; ardaksapieva73@mail.ru; 4 Faculty of Bioengineering and Bioinformatics, Lomonosov Moscow State University, Moscow 119234, Russia; 5 Translational Research Laboratory, All-Russian Scientific Research Institute of Medicinal and Aromatic Plants, Moscow 117216, Russia

**Keywords:** *Prunella vulgaris*, phytochemicals, extracts, antioxidant activity, antimicrobial activity, anti-inflammatory activity

## Abstract

**Background/Objectives**: This study compared five ultrasound-assisted extracts of *Prunella vulgaris* L. prepared with water or aqueous ethanol (30%, 50%, 70%, and 96%) and evaluated their phytochemical composition and preliminary antimicrobial, anti-inflammatory, brine-shrimp lethality, and antioxidant properties. **Methods**: Gallic acid and rosmarinic acid were quantified in all five extracts by a validated HPLC-UV method, with LC-ESI-MS used to support compound identification. Antimicrobial activity was evaluated by broth serial dilution and minimum inhibitory concentration (MIC) determination. Anti-inflammatory activity was assessed in a carrageenan-induced rat paw edema model. General toxicity screening was performed using the *Artemia salina* lethality assay. Antioxidant activity was evaluated by a ferricyanide reducing-power assay and the DPPH radical-scavenging assay. **Results**: Rosmarinic acid was detected in all extracts and was most abundant in PV70 (7.6%). PV70 showed MIC values of 12.5 μg/mL against *Staphylococcus aureus* and *Bacillus subtilis*. In the carrageenan model, PV70 produced 76.8% inhibition of paw-weight gain at 25 mg/kg, whereas diclofenac produced 66.6% inhibition at 10 mg/kg; this numerical comparison was descriptive and was not interpreted as evidence of superiority. The tested extracts caused low mortality of *Artemia salina* under the experimental conditions. **Conclusions**: The findings identify PV70 as a chemically distinct extract with notable activity in the screening models used. Because the extracts are multicomponent mixtures and mechanistic biomarkers were not measured, the observed biological effects cannot be attributed to rosmarinic acid alone. Further work is required to confirm mechanisms, safety, standardization, and efficacy.

## 1. Introduction

Conventional extraction procedures such as maceration and percolation remain widely used for medicinal-plant processing, but ultrasound-assisted extraction (UAE) can shorten extraction time and improve mass transfer while operating under comparatively mild conditions [1]. The efficiency of UAE depends on the plant matrix, solvent composition, extraction time, temperature, and acoustic conditions; therefore, extraction conditions should be optimized for each botanical material rather than assumed to be universally superior [2].

*Prunella vulgaris* L. (PV) grows in Kazakhstan, in the mountains of Central Asia, in Transcaucasia, the Baltic States, Moldova, Belarus, Ukraine, almost all European countries, Turkey, Iran, Pakistan, India, Japan, the northern and western provinces of China, North America, North Africa, and Australia. PV belongs to perennial herbaceous plants in the mint family (*Lamiaceae*) and is characterized by erect or ascending stems, serrated, smooth-edged or pinnate lobate, pinnate-incised or pinnate-divided leaves [3,4]. It is widely used in folk and traditional medicine and has been studied as a promising medicinal plant with anti-inflammatory, antihistamine, immunomodulating [5], antiviral, fungicidal [6], antioxidant [7] and antimicrobial [8] properties.

Cosmetic preparations containing active ingredients extracted from PV promote the biosynthesis of hyaluronic acid by skin epidermis cells. Among biologically active substances (BASs) found in PV are L-triterpenoids, steroids, flavonoids, coumarins, and higher fatty and unsaturated acids [9]. Essential oils obtained from the inflorescences are dominated by hexadecanoic acid (17.16%) and cyclo-1,6-decadienone (10.9%). It is important to consider potential variations in the quantitative content of active substances in different phenological stages of plant development and depending on whether the PV is sourced from an introduced environment or natural vegetation habitat [10].

*Prunella vulgaris* L. contains multiple classes of phytochemicals, including triterpenoids, phenolic acids, and flavonoids. Rosmarinic, caffeic, chlorogenic, and related hydroxycinnamic acids, together with flavonoids such as rutin, quercetin derivatives, and kaempferol derivatives, have been reported in the aerial parts. Because the abundance of these constituents varies with plant origin, phenological stage, extraction solvent, and analytical method, the biological activity of a crude extract should be interpreted as the result of its overall phytochemical composition rather than of a single marker compound.

Preliminary biological screening can help prioritize medicinal-plant extracts for more detailed pharmacological investigation. The brine shrimp lethality assay (BSLA) is a rapid screening model for general lethality but does not substitute for mammalian toxicology or a comprehensive safety assessment. Likewise, carrageenan-induced paw inflammation is a standard in vivo model for evaluating anti-inflammatory effects, but it does not by itself establish a molecular mechanism of action.

Polyphenol-rich plant extracts may influence inflammatory and oxidative processes through several pathways reported in the literature. However, a crude extract contains numerous constituents that may act additively, synergistically, or independently. Accordingly, mechanistic effects described for isolated phenolics should not be directly assigned to the whole *P. vulgaris* extract unless those mechanisms are experimentally demonstrated.

The present work was designed as a comparative, preliminary evaluation of five ultrasound-assisted *P. vulgaris* extracts rather than as a mechanistic or clinical efficacy study. The chemical and biological results are therefore interpreted within the limits of the assays performed.

Accordingly, this study aimed to compare five ultrasound-assisted *P. vulgaris* extracts prepared with water or 30%, 50%, 70%, and 96% ethanol, quantify selected phenolic markers, and evaluate antimicrobial activity, preliminary lethality in the BSLA model, carrageenan-induced anti-inflammatory activity, and in vitro antioxidant activity. Previous reports have examined *P. vulgaris* extraction and biological activity; the present study specifically compares extracts obtained with different ethanol–water compositions under the same UAE conditions.

## 2. Results

### 2.1. Quantitative HPLC-UV-ESI-MS/MS of Ultrasonic PV Extract

Five UAE extracts were prepared using water or aqueous ethanol (30%, 50%, 70%, and 96%). Extraction yields were 12.6% for PV96, 21.2% for PV70, 19.7% for PV50, 18.3% for PV30, and 16.8% for PVw (*p* < 0.001). The dried extracts were greenish-brown, hygroscopic materials with a characteristic odor. No claim of first-ever UAE of *P. vulgaris* is made because ultrasound-assisted extraction of this species has been reported previously.

**Figure 1:**
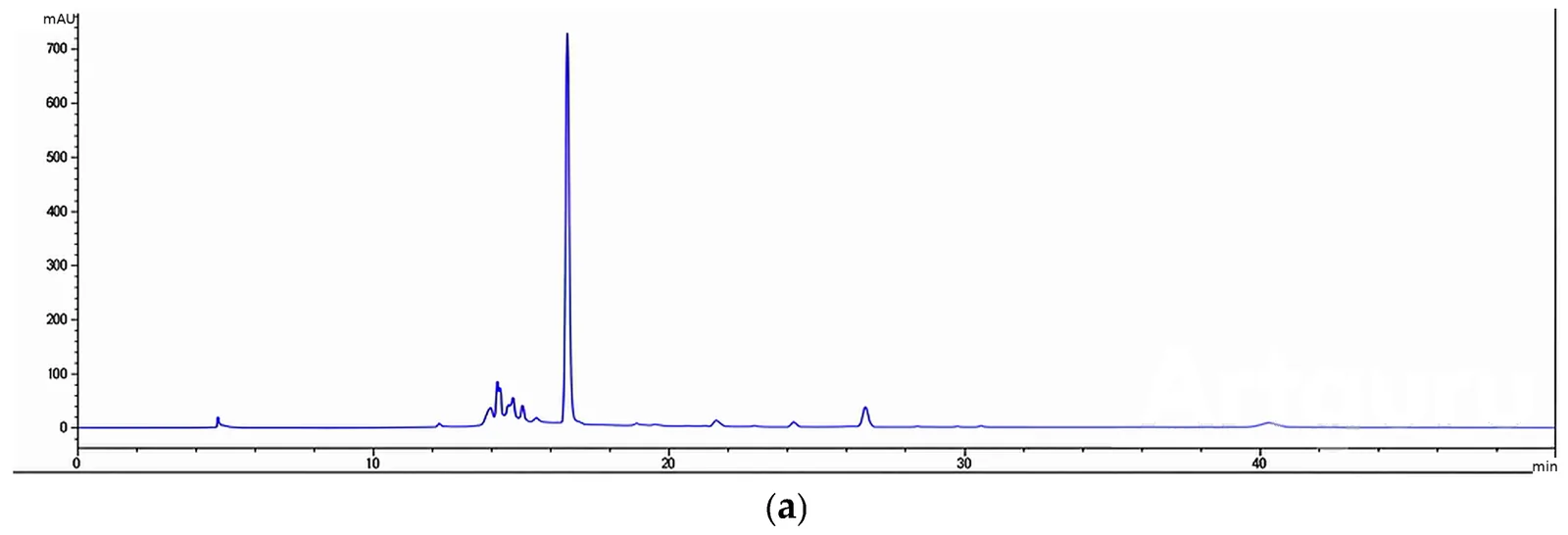

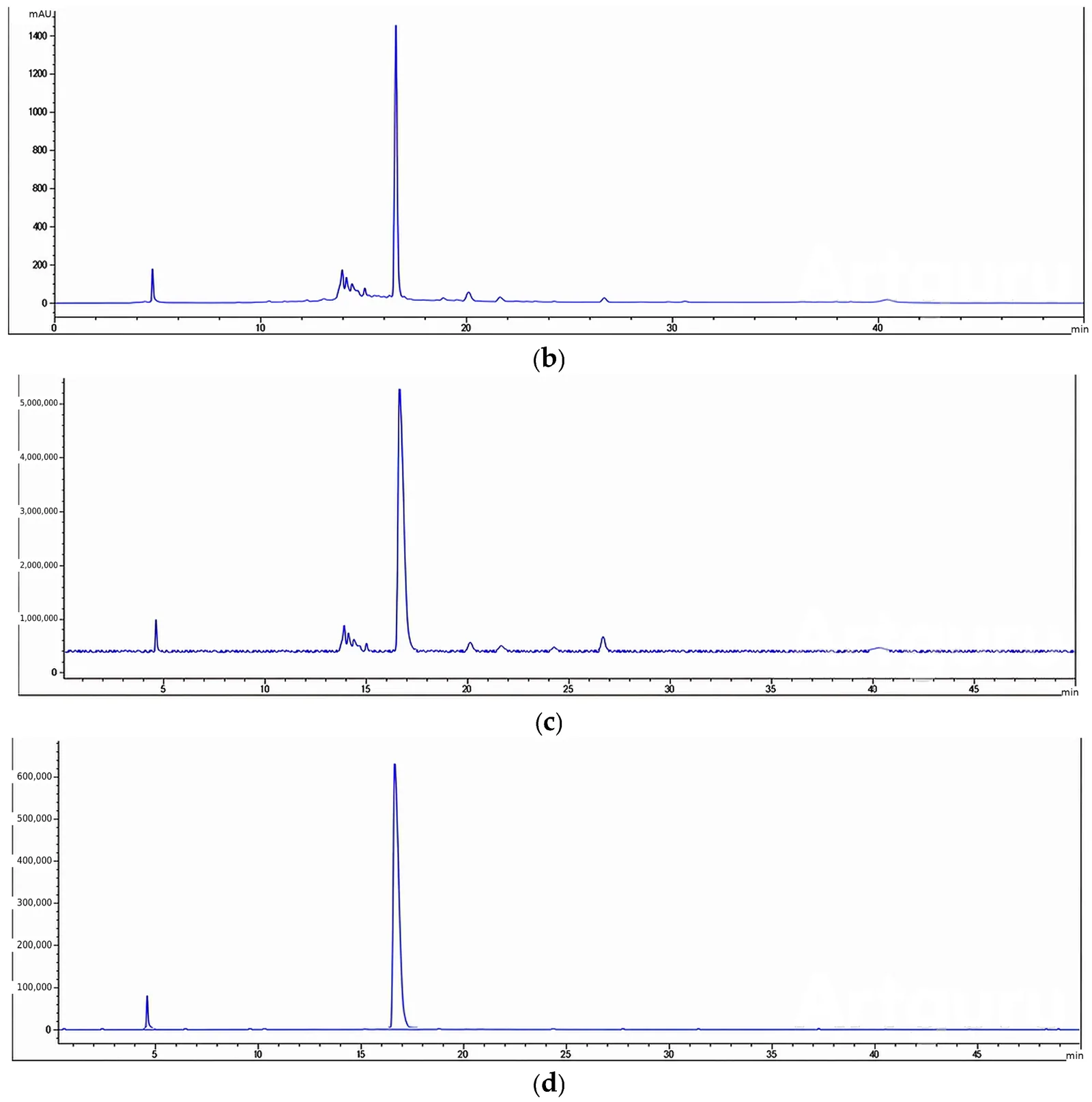
(**a**) HPLC-UV 360 nm, (**b**) HPLC-UV 280 nm, (**c**) the total ion chromatogram (TIC), (**d**) identified phenolic compounds of PV70 (EIC).

All five extracts (PV96, PV70, PV50, PV30, and PVw) were analyzed by HPLC-UV, and LC-ESI-MS data were used to support identification. Of the 20 reference phenolics screened, only gallic acid and rosmarinic acid produced analytical signals that could be reliably matched to authentic standards under the chromatographic conditions used (Table 1). The absence of a confirmed signal for the remaining standards indicates that they were not detected under these conditions; it does not establish their absolute absence from the plant matrix. PV70 contained the highest quantified level of rosmarinic acid among the five extracts Figure 1.

### 2.2. Antimicrobial Activity of the Extracts

The antimicrobial activity of the 5 PV ultrasonic extracts was measured by the serial dilution method and is shown in Table 2.

MIC was defined as the lowest extract concentration at which no visible microbial growth or turbidity was observed after incubation. Each determination was performed in at least three independent experimental replicates. Because plant extracts may themselves contribute color or turbidity, extract-containing blanks without microbial inoculum were included to aid visual interpretation. The solvent control did not inhibit microbial growth, and no growth was observed in the sterility control.

### 2.3. Anti-Inflammatory Activity

Carrageenan administration produced a marked increase in paw mass in the experimental animals. The anti-inflammatory results are summarized in Table 3.

Anti-inflammatory activity was evaluated in Wistar rats using carrageenan-induced paw inflammation. Paw mass was the experimental endpoint. At 4 h, PV70 significantly reduced paw-mass gain relative to the control (*p* < 0.05).

PV70 produced 76.8% inhibition of paw-weight gain at 25 mg/kg, whereas diclofenac produced 66.6% inhibition at 10 mg/kg under the experimental conditions used. The values are presented as a descriptive comparison only. Because the study was not designed or statistically powered as a superiority trial, the numerical difference should not be interpreted as evidence that PV70 is superior to diclofenac.

### 2.4. Toxicity

**Figure 2:**
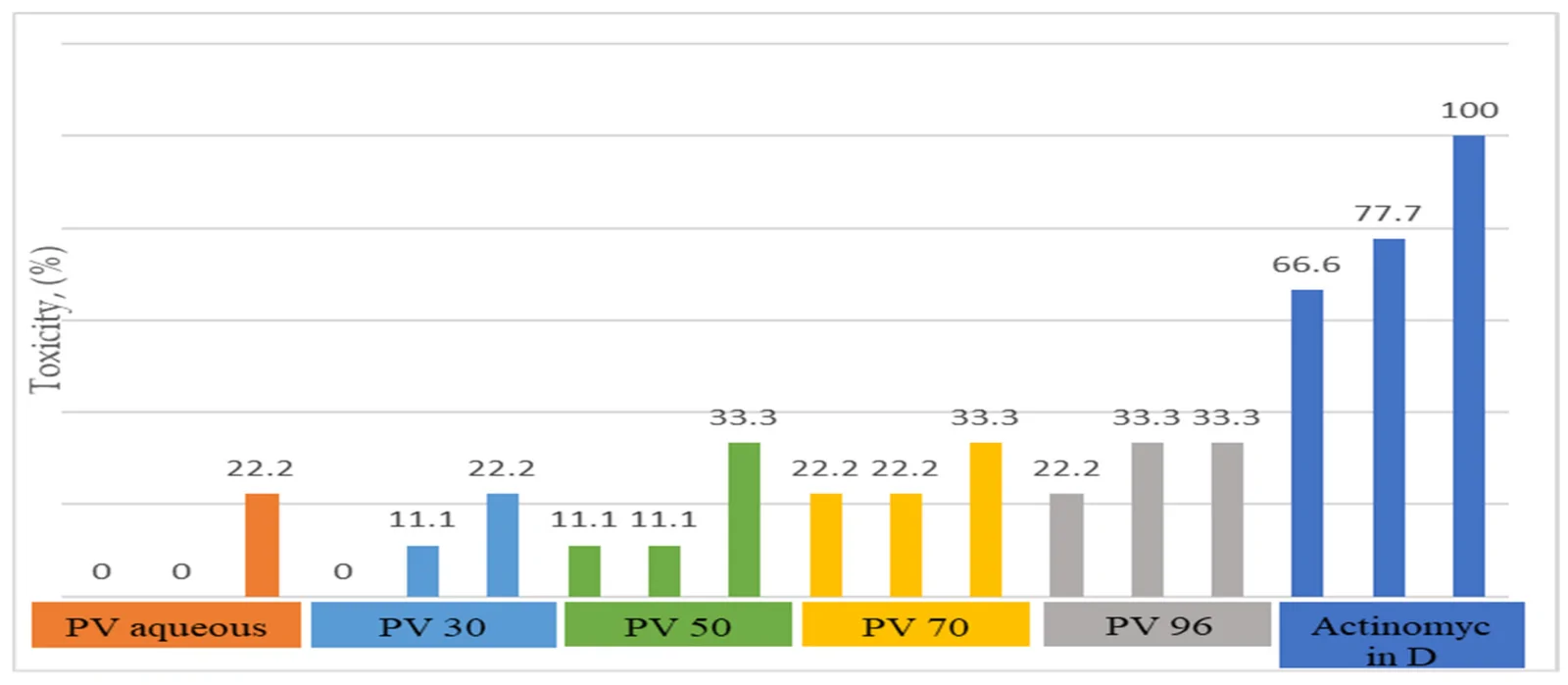
Toxicity of the studied samples PVw, PV30, PV50, PV70, and PV96 using BSLA.

The brine shrimp (*Artemia salina*) lethality bioassay (BSLA) was used for determination of toxicity of PV ultrasonic extracts. The data obtained during the BSLA bioassay are presented in Figure 2.

In the BSLA screening model, the tested extracts produced limited mortality under the experimental conditions. These findings should be interpreted only as preliminary lethality-screening results and not as evidence of systemic safety. Further toxicity studies in validated cellular and in vivo models are required before safety conclusions can be drawn.

### 2.5. Antioxidant Activity Using FRAP

The ferricyanide reducing-power assay was evaluated over the tested extract concentration range, with ascorbic acid used as the reference antioxidant. The numerical values originally reported as “FRAP IC_50_” are retained in Table 4 for transparency but are not interpreted as IC_50_ values because the assay, as performed, measured reducing power rather than 50% inhibition.

The ferricyanide assay used in this study measures reducing power through formation of a Prussian-blue complex detected at 700 nm. The original interpretation of a higher “FRAP IC_50_” as greater antioxidant activity was removed because this terminology is not appropriate for the assay as performed. The assay results should be interpreted as comparative reducing-power measurements under the stated experimental conditions.

### 2.6. Antioxidant Activity Using DPPH

All five extracts showed DPPH radical-scavenging activity within the concentration range tested. The results are reported as assay-derived IC_50_ values without using the scientifically ambiguous expression “high DPPH IC_50_”. Lower IC_50_ values correspond to greater radical-scavenging potency when IC_50_ is calculated from a valid concentration-response relationship (Table 5).

All five extracts showed measurable DPPH radical-scavenging activity under the tested conditions. Interpretation is based on the reported DPPH IC50 values, with lower IC50 indicating greater radical-scavenging potency when derived from an appropriate concentration–response relationship.

## 3. Discussion

*Prunella vulgaris* L. has a long history of traditional use and has been investigated for antioxidant [11], antiallergic [12], anti-inflammatory [13], and antimicrobial [14] properties. Phytochemical studies report several classes of constituents, including phenolic acids such as rosmarinic, ellagic, and caffeic acids, as well as triterpenoids and flavonoids [14]. Characterization of this multicomponent chemical profile is important when interpreting the biological activity of *P. vulgaris* extracts.

Previous studies have characterized the chemical constituents of different *P. vulgaris* plant parts. For example, HPLC analysis of a methanolic seed extract identified several major phenolic constituents, including caffeic acid (1.85 ± 0.05%) and rosmarinic acid (0.03 ± 0.00%) [15].

In a recent review [16], we highlighted several papers describing chemical composition of PV extracts (water, water/ethanol) using the HPLC method. RA, as well as oleanolic and ursolic acids, has been found in high quantities in PV spikes [17]. The authors indicated that the peak in the whole plant part of the PV extract responsible for the total phenol content determined by HPLC is RA [18]. Thus, in our study, HPLC analysis showed that PV70 ultrasonic extract contains a major content of rosmarinic acid of up to 7.6%, which contributes to a high anti-inflammatory effect. It is observed that all extracts contain rosmarinic acid in sufficient quantities from 0.95% to 7.6%, and an increase in the amount of water in the ethyl alcohol–water mixture can lead to a reduction in the concentration of the active substance as a result of the extraction process and, at the same time, the anti-inflammatory activity of the extracts decreases. A similar relationship can be seen in the study of toxicity, which decreases with increasing water content in a mixture of ethanol and water during the extraction process; for example, PV50—11.1%, PV70—22.2%, and PV96—22.2%.

Previous studies have reported antimicrobial activity for *P. vulgaris* extracts prepared with several solvents. In the present study, none of the five extracts inhibited *Escherichia coli* or *Pseudomonas aeruginosa* at the concentrations tested. PV70 showed MIC values of 12.5 μg/mL against *Staphylococcus aureus* and *Bacillus subtilis* and 50 μg/mL against *Candida albicans*. The MIC of PV70 against B. subtilis was numerically identical to the ceftriaxone MIC obtained under these experimental conditions; however, this is a descriptive analytical comparison and should not be interpreted as equivalent antimicrobial efficacy because PV70 is a complex crude extract, whereas ceftriaxone is a purified antimicrobial drug.

Previous studies have also reported the MIC of PV extracts against different bacterial pathogens. Ozbek et al. found that the MIC of ethyl acetate PV extract against *Staphylococcus aureus*, *Escherichia coli*, and *Pseudomonas aeruginosa* was 6.25 μg/mL, while that of hexane PV extract against *Escherichia coli* was 6.25 μg/mL and against *Pseudomonas aeruginosa* was 1.56 μg/mL [19]. In our study, neither PV extract showed antimicrobial activity against *Pseudomonas aeruginosa* or *Escherichia coli*. Another study [20,21] demonstrated that aqueous and ethanolic extracts of PV showed antimicrobial activities against *Escherichia coli* of 2 and 1.33 μg/mL, respectively. The MIC values for methanolic extracts vary in studies; for instance, in one study, the MIC for *Staphylococcus aureus* and *Escherichia coli* was 100 μg/mL [22], whereas a different study showed values for *Staphylococcus aureus* of 25 μg/mL and *Escherichia coli* of 200 μg/mL [23]. A final study indicated that the MIC of aqueous extract of PV was 50 μg/mL, while in our study, the MIC for aqueous extract was not determined.

Carrageenan-induced rat paw edema is the most commonly used and convenient approach for assessing the anti-inflammatory activity of substances. The anti-inflammatory activity of 50% aqueous and methanolic extracts obtained from floral spikes of PV from India was previously investigated [24]; both extracts showed anti-inflammatory activity with a percentage of inhibition of inflammation of 28.9% for aqueous extracts and 30.8% for methanolic extracts, while we obtained a result of 45% for our 50% extract. The differences between our results and those obtained by Gazanfar et al. can be attributed to the use of different plant parts and the location of growth, which influence the chemical composition of extracts [24].

A previous study provided evidence that PV extracts could cause inhibition of nitric oxide and prostaglandin E2 production, as well as inducible mRNA expression of nitric oxide synthase, cyclooxygenase-2, and tumor necrosis factor-α [25]. Ryu et al. reported that PV herbs have a strong anti-inflammatory activity due to the presence of triterpenes, particularly of ursolic acid and 2α-hydroxyursolic acid [26].

In the present study, the anti-inflammatory activity of five ultrasound-assisted *P. vulgaris* extracts was evaluated using carrageenan-induced paw inflammation in Wistar rats. PV70 produced 76.8% inhibition of paw-mass gain at 4 h. This result demonstrates activity in the model but does not establish a specific molecular mechanism.

Thus, it can be assumed that the pronounced inhibitory effect of PV70 on inflammation caused by carrageenan may be due to inhibition of the COX-2, leading to inhibition of prostaglandin E2 synthesis. This is explained by the high content of RA in PV70, which is confirmed by literature data showing that RA inhibits COX-2 expression, partially explaining the anti-inflammatory activity of this sample [13].

PV70 showed the largest inhibition of paw-weight gain among the tested extracts. Its inhibition value (76.8%) was numerically higher than that observed with diclofenac (66.6%), but the experiment did not provide a direct statistical test designed to demonstrate superiority. In addition, the tested doses differed (25 mg/kg for PV70 and 10 mg/kg for diclofenac); therefore, no claim of superiority is made.

Currently, many chemotherapeutic drugs are derived from natural sources and used against a variety of diseases. Unfortunately, most of these compounds often exhibit systemic toxicity and side effects.

Previously, the toxicity of the extracts of spikes of *P. vulgaris* var. lilacina from different solvent fractions was assessed using a tetrazolium-based colorimetric assay (MTT assay) against human colon carcinoma (HT-29), human breast carcinoma (MCF-7) and human liver carcinoma (HepG2) cell lines [27]. The results showed that some compounds derived from *P. vulgaris* var. lilacina at the concentration of 100 μM have moderate toxicity against the HT-29 cell line, but other compounds have weak toxicity against HT-29, MCF-7 and HepG2 cell lines. In another study [28], the toxicity of aerial parts of *P. vulgaris* var. lilacina using a sulforhodamin B bioassay (SRB) against non-small cell lung adenocarcinoma (A549), ovarian cancer (SK-OV-3), skin melanoma (SK-MEL-2) and colon cancer (HCT 15) tumor cell lines was reported.

To assess the tolerability of selected potentially bioactive specimens, *Artemia salina* is commonly used as a model in mortality studies. BSLA seems to be the quickest, easiest-to-use, and most cost-effective method among the available options [29]. This method provides a convenient starting point for toxicity investigations, including general toxicity screening of synthetic, semi-synthetic and natural products.

In the present investigation, the toxicity of five PV extracts using BSLA was observed. Based on the results of the experiment, it was found that PV50, PV70, and PV96 at concentrations of 1, 10 and 100 μg/mL exhibit weak toxicity, so the mortality of shrimps when using PV50 is 11.1%, 11.1% and 33.3%, respectively; when using PV70—22.2%, 22.2% and 33.3%, respectively; and when using PV96—22.2%, 33.3% and 33.3%, respectively.

PVw shows toxicity at a concentration of 100 μg/mL of 22.2%. PV30 exhibits toxicity at concentrations of 10 and 100 µg/mL of 11.1% and 22.2%, respectively.

In summary, PV50, PV70, and PV96 at concentrations of 1, 10 and 100 µg/mL, PV30 at concentrations of 10 and 100 µg/mL, and PVw at a concentration of 100 µg/mL show weak toxicity by *A. salina* (Leach).

In BSLA, mortality of A. salina exposed to the tested extracts ranged from approximately 10% to 33% under the experimental conditions. These results are described as showing low mortality in this preliminary screening model and should not be interpreted as evidence that the extracts or products derived from them are safe.

It can be concluded that all tested extracts that do not show general toxicity can be considered as candidates for further studies of activity and toxicity in vitro on selected cell lines and in vivo on laboratory animals. If the absence of toxicity is demonstrated in various models, it will be possible to develop new bioactive agents and in-depth studies of pharmacological properties.

Antioxidant activity was evaluated using the DPPH radical-scavenging assay and a ferricyanide reducing-power assay [30]. The original manuscript incorrectly referred to the latter results as “FRAP IC_50_” and interpreted higher values as greater activity. This interpretation has been removed. DPPH IC_50_ values are reported as quantitative descriptors of radical-scavenging activity, whereas the ferricyanide results are discussed as reducing-power measurements. Differences from published studies may reflect plant origin, plant part, phenological stage, extraction solvent, extraction conditions, and assay methodology [31].

The biological activity observed for PV70 may be associated with its overall phytochemical composition, including its relatively high rosmarinic acid content. However, the contribution of individual constituents was not independently established in this study. Published work has reported antioxidant and anti-inflammatory effects for rosmarinic acid, but those literature data should not be interpreted as proof that rosmarinic acid alone caused the effects observed for the crude PV70 extract [32,33,34,35].

The present study has several limitations. The biological findings are based on a limited panel of experimental models, and the *Artemia salina* assay should be considered a preliminary toxicity-screening approach. In addition, inflammatory biomarkers were not directly measured; therefore, the molecular mechanisms discussed for the observed anti-inflammatory effects remain to be experimentally confirmed. Furthermore, bioactivity-guided fractionation was not performed, and the biological effects of the crude extracts cannot be attributed to individual constituents. Further studies are required to confirm these findings and clarify the compounds and mechanisms responsible for the observed activities. In summary, based on the results above, it is evident that PV has great potential as a promising natural source of BASs, especially RA, and can be utilized in pharmaceutical production.

## 4. Materials and Methods

### 4.1. Chemicals and Drugs

Diclofenac sodium (Merkle, Hamburg, Germany); isotonic sodium chloride solution, α-carrageenan, potassium hexacyanoferrate (III), trichloroacetic acid, acetonitrile, phosphoric acid, disodium hydrogen phosphate, actinomycin D, chlorogenic, gallic, caffeic, trans-ferulic, rosmarinic, oleanolic, ursolic acids, and flavonoid quercetin were purchased from Sigma-Aldrich Co. (St. Louis, MO, USA); *A. salina* eggs, ethyl alcohol 96%, ceftriaxone (Sintez, Kurgan, Russia); nystatin (Biosintez, Penza, Russia); acetonitrile (ACN) for HPLC (≥99, 9%, Sigma-Aldrich, Saint-Quentin-Fallavier, France); formic acid (99–100%, AnalaR NORMAPUR^®^, VWR Chemicals, Rosny-sous-Bois, France). Highly purified water was prepared using a Milli-Q water purification system (Millipore, Molsheim, France). Twenty selected phenolic compounds and standards for HPLC (gallic acid, caffeic acid, chlorogenic acid, ferulic acid, p-coumaric acid, rosmarinic acid, cinnamic acid, catechin, epicatechin, naringin, rutin, luteolin-7-O-glucoside, quercetin-3-glucoside, dihydroquercetin, myricetin, quercetin, naringenin, apigenin, luteolin, kaempferol) were purchased from Sigma-Aldrich (St. Louis, MO, USA).

### 4.2. Plant Material

The study material consisted of the aerial parts (leaves, inflorescences, and stems) of *Prunella vulgaris* L. collected in the Ulytau Mountains, Ulytau Region, Republic of Kazakhstan (47°17′5.0914″ N, 68°21′11.9862″ E; 365 m above sea level) during the flowering stage in the second ten-day period of July 2022. Species identification was performed by Prof. Margarita Ishmuratova (Faculty of Biology and Geography, Karaganda Buketov University), and a voucher specimen (QAR04149QAR) was deposited at the Institute of Botany, Karaganda Buketov University, Kazakhstan.

### 4.3. Animals

Seventy adult male Wistar rats weighing 240–260 g were used. Animals were maintained in the vivarium of Karaganda Medical University at 22 ± 2 °C under a natural light cycle, with a standard diet and water available ad libitum. Before the experiment, animals underwent a three-day quarantine/acclimatization period for observation of general health and adaptation to housing conditions. Animals were randomly allocated to seven experimental groups (*n* = 10 per group), and each animal was used once. No investigator blinding procedure was documented in the source experimental records. All procedures complied with the National Institutes of Health Guide for the Care and Use of Laboratory Animals and were approved by the Institutional Animal Ethical Committee of Karaganda Medical University (Approval No. 60; Protocol No. 18; 14 April 2021).

### 4.4. Preparations of Plant Extract Using UAE

The same aerial plant material collected in July 2022, as described in Section 4.2, was used for UAE.

Air-dried *P. vulgaris* aerial material was milled to 3–5 mm. A 20.0 g portion was extracted with 400 mL of water or 30%, 50%, 70%, or 96% ethanol (solid-to-liquid ratio 1:20, *w*/*v*). UAE was performed without pre-soaking in a Stegler 3DT ultrasonic bath (Stegler, Kunshan, China; 3 L capacity, 40 kHz, 120 W) at 20–22 °C for 30 min. The liquid phase was decanted, and the extraction was repeated once under the same conditions. The resulting extracts were designated PVw, PV30, PV50, PV70, and PV96 according to the extraction solvent.

The two liquid extracts obtained for each solvent were combined and filtered through paper. Solvent was removed under reduced pressure at 50 °C using a rotary evaporator, followed by removal of residual solvent in a water bath at 60 °C. Extraction yield was calculated as the mass of dried extract divided by the initial mass of dry plant material × 100%. The extraction yields used in the comparative analysis were 12.6% (PV96), 21.2% (PV70), 19.7% (PV50), 18.3% (PV30), and 16.8% (PVw).

### 4.5. Quantitative HPLC-UV-ESI-MS/MS Analysis of Ultrasonic Extract

All five extracts (PV96, PV70, PV50, PV30, and PVw) underwent quantitative HPLC-UV analysis for gallic acid and rosmarinic acid. LC-ESI-MS data were used as orthogonal evidence to support identification. Twenty phenolic reference standards were screened under the analytical conditions described below; only gallic acid and rosmarinic acid yielded signals that were reliably confirmed against authentic standards and were therefore quantified.

The analysis was conducted using an “Agilent 1260 Infinity HPLC system” liquid chromatograph (Agilent Technologies, Santa Clara, CA, USA), equipped with a G1311C 1260 Pump VL, autosampler G1329B 1260 ALS, thermostatted column compartment G1316A 1260 TCC, variable wavelength detector G1314C 1260 VWD VL+, and mass-spectrometer G6130A Quadrupole LC-MS/MS. The system was controlled by the Windows NT- based ChemStation software (Agilent ChemStation B.04.03 SP1).

Chromatographic separation was performed on a Zorbax Eclipse Plus C18 column (150 × 4.6 mm, 3.5 μm; Agilent Technologies, Santa Clara, CA, USA). Mobile phase A was 2.5% (*v*/*v*) formic acid in water and mobile phase B was 2.5% (*v*/*v*) formic acid in acetonitrile. The gradient was: 0.00 min, 3% B; 7.00 min, 20% B; 7.10 min, 30% B; 27.00 min, 40% B; 35.00 min, 50% B; 35.10 min, 20% B; and 40.00 min, 3% B. The flow rate was 0.4 mL/min, the column temperature was 30 °C, and the injection volume was 20 μL. Samples and standards were prepared in acetonitrile/water (1:1, *v*/*v*). UV detection was performed at 280 and 360 nm. Electrospray ionization was operated in negative-ion mode (capillary temperature 350 °C; N2 drying gas 8 L/min; nebulizer pressure 45 psi). The previous MRM/SIM terminology was removed because it did not correspond to the acquisition mode used with the G6130A instrument in this study. Extracted-ion chromatograms (EICs) were generated from the full-ion-current data for the target *m*/*z* values.

Compound identity was assessed by agreement of retention time with authentic standards and by comparison of the observed mass spectra/fragment ions with those of reference standards and published data. In negative-ion mode, gallic acid showed [M − H^−^] − at *m*/*z* 169 with fragments at *m*/*z* 125, 107, and 79, whereas rosmarinic acid showed [M − H^−^] − at *m*/*z* 359 with fragments at *m*/*z* 197, 179, and 161. Quantification was performed by the external-standard method. Each analysis was performed in triplicate.

The analytical method used for the quantitative determination of gallic acid and rosmarinic acid was evaluated for linearity and range, limits of detection (LOD) and quantification (LOQ), precision, accuracy, selectivity, robustness, and solution stability. Detailed validation procedures and results are provided in the Supplementary Materials (Tables S1–S10).

### 4.6. Antimicrobial Activity Assay

The antimicrobial activity of ultrasonic extracts PV96, PV70, PV50, PV30, and PVw was studied in relation to strains of Gram-positive bacteria including *Staphylococcus aureus* (ATCC 6538) and *Bacillus subtilis* (ATCC 6633), and Gram-negative bacteria such as *Escherichia coli* (ATCC 25922), *Pseudomonas aeruginosa* (ATCC 27853) and the yeast fungus *Candida albicans* (ATCC 10231) by the serial dilution method with determination of the minimum inhibitory concentration (MIC) [36]. The antibacterial drug ceftriaxone and the antifungal drug nystatin were used for comparison.

MIC was determined by broth serial dilution. Test-strain suspensions were adjusted to approximately 10^6^ CFU/mL. Extract concentrations ranged from 1.56 to 50 μg/mL. After inoculation and incubation (24 h at 37 °C for bacteria; 48 h at 30 °C for *C. albicans*), MIC was defined as the lowest concentration with no visible microbial growth or turbidity. Each assay was performed at least in triplicate and was read by one investigator. Because colored plant extracts can interfere with visual turbidity assessment, extract-containing sample blanks without microbial inoculum were used to aid interpretation. A solvent control containing the corresponding amount of 40% ethanol without extract showed no growth-inhibitory effect. A sterility control containing uninoculated nutrient medium showed no microbial growth. MBC and MFC were not determined.

### 4.7. Anti-Inflammatory Activity Using Carrageenan-Induced Rat Paw Edema

The in vivo experimental procedures were conducted in accordance with the “Regulations of the European Convention for the Protection of Vertebrate Animals Used for Experimental and Other Scientific Purposes” and in accordance with the requirements for the study of new pharmacological substances.

Paw inflammation was induced by subplantar injection of 0.1 mL of carrageenan suspension in 1% NaCl into the left hind paw. The 70 Wistar rats were randomly allocated to seven groups (*n* = 10): vehicle control, PV96, PV30, PV50, PVw, PV70, and diclofenac. Each *P. vulgaris* extract was administered intragastrically by oral gavage at 25 mg/kg, and diclofenac sodium was administered intragastrically by oral gavage at 10 mg/kg. Paw mass was the endpoint measured in the experiment. Paw-mass gain was calculated as the difference between the mass of the edematous paw and the corresponding intact paw at the 4 h peak inflammatory response. Inhibition was expressed relative to the vehicle control. No formal a priori power calculation was documented; the sample size was 10 animals per group according to the experimental protocol.

Anti-inflammatory activity was expressed as the percentage of edema inhibition at the peak of inflammation compared to the control. The percentage inhibition of the inflammation was determined using the following formula:

%I = (dc − dt) × 100%/dc(1)

where dt is the paw-mass increase in the treated group, dc is the paw-mass increase in the control group, and I is the percentage inhibition of inflammation.

### 4.8. Preliminary Assessment of Toxicity Using Brine Shrimp Lethality Assay (BSLA)

The shrimps were reared by immersing eggs of marine crustaceans *A. salina* (Leach) in artificial seawater and then incubated for 48 h at a temperature of 37 °C.

Preliminary general lethality was evaluated using the brine shrimp lethality assay (BSLA). Extracts were tested at 1, 10, and 100 μg/mL in triplicate, with 10 two-day-old *Artemia salina* nauplii per vial and a 24 h exposure. Actinomycin D was used as a positive control because of its established cytotoxic activity and was tested at the same concentrations (1, 10, and 100 μg/mL) to confirm responsiveness of the assay. The corresponding ethanol vehicle served as the negative control. BSLA results are described as mortality in this model and are not interpreted as a comprehensive measure of mammalian toxicity or safety.

A portion of ultrasonic extracts was dissolved in 2 mL of ethanol; then, 500 µL (3 parallels), 50 µL (3 parallels), and 5 µL (3 parallels) were taken from this solution. After evaporation of the ethanol, 5 mL of artificial sea water was added to each vial. Thus, if the initial sample weight was 2 mg, then the final sample concentrations were 100 µg/mL, 10 µg/mL, and 1 µg/mL, respectively, of each concentration in 3 repetitions. In each vial, 10 active shrimps, 2 days old, were planted using a Pasteur pipette. Subsequently, all vials were left at room temperature in the light for 24 h. After this period, the surviving and dead shrimps were counted. Actinomycin D was used as the control. The control was ethanol in equivolume amounts.

To determine the toxicity, the percentage of dead shrimps (A, %) was calculated compared to the control using the formula:

%A = (H_k_ − H_o_)/H_k_ ∗ 100% (2)

where H_k_ represents the number of surviving shrimps in the control; H_o_ is the number of surviving shrimps in the experiment.

### 4.9. Antioxidant Capacity

Ferricyanide reducing-power assay. The reducing capacity of the extracts was evaluated using the potassium ferricyanide method [37]. This assay measures the reduction of Fe^3+^ and subsequent formation of a colored complex detected at 700 nm. Because the procedure used here is not the conventional TPTZ-based FRAP assay, the section and interpretation were revised accordingly and the term “FRAP IC_50_” was removed.

For each extract, 1 mL of sample solution (0–1 mg/mL) was mixed with 2.5 mL phosphate buffer (0.2 M, pH 6.6) and 2.5 mL of 1% potassium ferricyanide solution. After incubation at 50 °C for 25 min, 2.5 mL of 10% trichloroacetic acid was added. The mixture was centrifuged, and 2.5 mL of the supernatant was mixed with 2.5 mL distilled water and 0.5 mL of 0.1% FeCl3. Absorbance was measured at 700 nm. Higher absorbance reflects greater reducing power under the assay conditions.

[Fe (CN)6]^3−^ + ArOH → [Fe (CN)^6^]^4−^ + ArO + H^+^

[Fe (CN)6]^4−^ + Fe^3+^ + K^+^ → K Fe [Fe (CN)^6^]   λ = 700 nm.

The procedure was conducted following the method described by Kirillov et al. [38]: 2.5 mL of phosphate buffer (0.2 M, pH 6.6) and 2.5 mL of 1% solution of potassium hexacyanoferrate (III) were added to 1 mL of the test extracts with concentrations ranging 0–1 mg/mL. The reaction mixture was incubated for 25 min at 50 °C; to stop the reaction, 2.5 mL of 10% solution of trichloroacetic acid was added. The mixture was centrifuged for 3 min (1.5 revolutions per minute). Then, 2.5 mL was skimmed and mixed with 2.5 mL of distilled water and 0.5 mL 0.1% FeCl^3^. The optical density (OD) was measured at λ = 700 nm.

### 4.10. DPPH Radical Scavenging Assay

The DPPH radical-scavenging assay was used to evaluate free-radical scavenging activity according to the method of Blois. A 0.1 mL aliquot of each extract solution (0.25, 0.50, 0.75, and 1.00 mg/mL) was added to 3 mL of 6 × 10^−5^ M DPPH solution [39]. The mixtures were incubated in the dark for 30 min and absorbance was measured at 520 nm. Antioxidant activity (AOA, %) was calculated relative to the DPPH control. IC_50_ values, where reported, represent the concentration estimated to produce 50% DPPH radical scavenging.

AOA = A_0_ − A_t_/A_0_ × 100(%) (3)

where A_0_ is the value of the OD of the control sample; At is the value of the OD at a given concentration of the test solution.

### 4.11. Statistical Analysis

Descriptive antimicrobial results were summarized from at least three replicate determinations. HPLC validation experiments were performed in triplicate as specified for each parameter.

Statistical analyses were performed using STATISTICA software (version 8.0; StatSoft Inc., Tulsa, OK, USA). Animal data are presented as mean ± SD, with *n* = 10 biological replicates per group. The original analysis used the Mann–Whitney U-test for comparisons with the control group at *p* < 0.05. The available revision materials do not contain individual animal-level observations or a documented normality test; therefore, a valid seven-group omnibus re-analysis (ANOVA with an appropriate post hoc test or Kruskal–Wallis test with multiplicity-corrected post hoc comparisons, depending on distributional assumptions) cannot be reconstructed. Accordingly, no claim of superiority between PV70 and diclofenac is made, and this statistical limitation is stated explicitly.

## 5. Conclusions

This study provides a preliminary comparative characterization of five ultrasound-assisted *P. vulgaris* extracts prepared with different ethanol–water compositions. PV70 contained the highest measured rosmarinic acid level and showed notable activity in the antimicrobial and carrageenan-induced inflammation screening models. These observations represent associations at the crude-extract level and do not demonstrate that rosmarinic acid alone determines the biological effects. The *Artemia salina* results indicate low mortality in this preliminary model rather than established safety. The antioxidant findings should be interpreted according to the specific DPPH radical-scavenging and ferricyanide reducing-power assays used. Overall, the results support further investigation of standardized *P. vulgaris* extracts, including bioactivity-guided fractionation, expanded antimicrobial testing, validated mechanistic studies, formal toxicological assessment, and adequately powered efficacy experiments.

The results of these studies suggest that PV is a potential source of a natural agent. The findings obtained allow us to emphasize the potential use of PV herbs as a source of non-essential and essential natural biologically active substances.

## Figures and Tables

**Table 1:** Identification and content of phenolic compounds of ultrasonic extracts from *Prunella vulgaris* L.

Peak	Retention Time (min)	M − H^−^ (*m*/*z*)	Compound Identity	Height, Dry Extract (%)				
				PV96	PV70	PV50	PV30	PVw
1	4.832	169	Gallic acid	0.74 ± 0.22	0.99 ± 0.1	0.4 ± 0.18	0.17 ± 0.11	0.12 ± 0.08
2	16.786	359	Rosmarinic acid	5.86 ± 0.74	7.6 ± 0.9	5.77 ± 0.86	0.22 ± 0.42	0.95 ± 0.20

**Table 2:** Minimum inhibitory concentrations (μg/mL) of the PV ultrasonic extracts.

Sample Cipher	*Staphylococcus* *aureus* ATCC 6538	*Bacillus* *subtilis* ATCC 6633	*Escherichia* *coli* ATCC 25922	*Pseudomonas* *aeruginosa* ATCC 27853	*Candida* *albicans* ATCC 10231
PV96	25	50	-	-	-
PV70	12.5	12.5	-	-	50
PV50	-	-	-	-	-
PV30	-	-	-	-	-
PVw	-	-	-	-	-
Ceftriaxone	6.3	12.5	6.3	12.5	-
Nystatin	-	-	-	-	12.5

**Table 3:** Anti-inflammatory activity of ultrasound-assisted extracts in the carrageenan-induced rat paw inflammation model.

Substances	Mass of the Intact Paw (g)	Mass of the Edematous Paw (g)	Weight Gain (g)	Weight Gain (%)	Inhibitory Effect (%)
Control	113.4 ± 2.6	180.7 ± 5.7	67.3 ± 3.5	59.3 ± 2.2	—
PV96	115.3 ± 3.7	161.6 ± 4.8	46.3 ± 4.0	40.1 ± 3.5	31.2
PV30	118.6 ± 2.4	154.4 ± 3.5	35.8 ± 1.9	30.2 ± 3.8	46.8
PV50	119.0 ± 2.2	156.7 ± 4.3	37.7 ± 4.0	31.2 ± 2.0	44.0
PVw	117.1 ± 1.9	148.4 ± 2.6	31.3 ± 2.6 *	26.7 ± 4.2	53.5
PV70	116.6 ± 1.3	132.2 ± 2.0 *	15.6 ± 1.5 *	13.4 ± 3.5 *	76.8
Diclofenac	120.2 ± 3.2	142.7 ± 3.4 *	22.5 ± 1.3 *	18.7 ± 1.6 *	66.6

*—*p* < 0.05 compared to control.

**Table 4:** Half-maximal inhibitory concentration (IC_50_) of *Prunella vulgaris* extracts determined using the FRAP assay.

No.	Sample	Reported Reducing-Power Assay Value
1	PV96	0.71 ± 0.03
2	PV70	0.83 ± 0.02
3	PV50	0.67 ± 0.02
4	PV30	0.48 ± 0.03
5	PVw	0.55 ± 0.02
6	Control	0.79 ± 0.02

**Table 5:** Half-maximal inhibitory concentration (IC_50_) of *Prunella vulgaris* extracts determined using the DPPH assay.

No.	Sample	DPPH IC50 (μg/mL)
1	PV96	36.5
2	PV70	41.3
3	PV50	40.7
4	PV30	48.1
5	PVw	46.2

## Data Availability

The original contributions presented in this study are included in the article/Supplementary Materials. Further inquiries can be directed to the corresponding author(s).

## Supplementary Materials

The following supporting information can be downloaded at: https://www.mdpi.com/article/10.3390/ph19101646/s1. Table S1. Linearity range, regression equation, coefficient of determination, and correlation coefficient for GA and RA. Table S2. Slope and slope RSD for GA and RA calibration curves. Table S3. Intraday and interday precision for gallic acid. Table S4. Intraday and interday precision for rosmarinic acid. Table S5. Accuracy data for gallic acid. Table S6. Accuracy data for rosmarinic acid. Table S7. Robustness data for gallic acid. Table S8. Robustness data for rosmarinic acid. Table S9. Stability of gallic acid solutions after 5 and 7 days at −20 °C, 4 °C, and room temperature. Table S10. Stability of rosmarinic acid solutions after 5 and 7 days at −20 °C, 4 °C, and room temperature. Table S11. LC-MS characteristics used to support identification of gallic acid and rosmarinic acid. Figure S1. Representative HPLC-UV chromatograms at 360 and 280 nm, total ion chromatogram (TIC), and extracted-ion chromatogram (EIC) used to support identification of phenolic compounds in PV70. Refs. [40,41,42] were cited in the Supplementary Materials.

## Abbreviations

The following abbreviations are used in this manuscript:

AAE	Ascorbic Acid Equivalent
ACN	Acetonitrile
AOA	Antioxidant Activity
ATCC	American Type Culture Collection
BAS	Biologically Active Substances
BSLA	Brine Shrimp Lethality Assay
CFU	Colony Forming Units
COX-2	Cyclooxygenase-2
DPPH	2,2-Diphenyl-1-picrylhydrazyl
ESI-MS/MS	Electrospray Ionization Tandem Mass Spectrometry
EIC	Extracted-Ion Chromatogram
HPLC	High-Performance Liquid Chromatography
IC_50_	Half Maximal Inhibitory Concentration
MIC	Minimum Inhibitory Concentration
NSAIDs	Non-Steroidal Anti-Inflammatory Drugs
PV	*Prunella vulgaris* L.
RA	Rosmarinic Acid
TIC	Total Ion Chromatogram
UAE	Ultrasound-Assisted Extraction
UV	Ultraviolet Detector
