# Supplementary material for: Antimicrobial, Anti-Inflammatory, General Toxicity and Antioxidant Activities of Ultrasound-Assisted Extracts from Prunella vulgaris L
Source: Pharmaceuticals (Basel). 2026 Oct 19;19(10):1646. doi: 10.3390/ph19101646 (PMC13708712; doi:10.3390/ph19101646)
Supplement: Supplementary file 1 [file pharmaceuticals-19-01646-s001.zip › pharmaceuticals-4546535-supplementary.pdf]

Supplementary Materials

Antimicrobial, anti-inflammatory, general toxicity and antioxidant activities of ultrasound-assisted extracts from Prunella vulgaris L.

S1. HPLC-UV Method Validation for Gallic Acid and Rosmarinic Acid

The quantitative HPLC-UV method for gallic acid (GA) and rosmarinic acid (RA) was evaluated for linearity and range, LOD and LOQ, precision, accuracy, selectivity, robustness, and solution stability. The validation data below are based on the authors' supplied analytical records.

S1.1. Linearity and range

Seven calibration levels (0.2, 2.0, 10, 90, 120, 180, and 250 μg/mL) were analyzed in triplicate on different days. Slope RSD was calculated as 100 × sm/m, where sm is the standard deviation of the slope and m is the mean slope.

S1.2. LOD and LOQ

LOD and LOQ were calculated from the calibration slope and the standard deviation of the analytical response obtained from 10 blank samples analyzed in triplicate: LOD = 3.3ẟ/s and LOQ = 10ẟ/s.

S1.3. Precision

Intraday and interday precision were evaluated at 0.25, 0.65, 5, 120, and 180 μg/mL in triplicate and expressed as RSD (%).

S1.4. Accuracy

Accuracy was evaluated by back-calculation of known GA and RA standard concentrations and expressed as obtained concentration / nominal concentration × 100.

S1.5. Selectivity

A model mixture containing caffeic, gallic, chlorogenic, p-coumaric, rosmarinic, and cinnamic acids was analyzed to verify chromatographic separation of GA and RA from related phenolic acids.

S1.6. Robustness

Robustness was explored by small changes in mobile-phase formic-acid content and flow rate, with peak area and retention time compared with the optimized method.

S1.7. Stability

GA and RA solutions at 0.4, 4, and 120 μg/mL were evaluated after storage at −20 °C, 4 °C, and room temperature for 5 and 7 days.

Table S1. Linearity range, regression equation, coefficient of determination, and correlation coefficient for GA and RA.

**Compound - Calibration Curve**	**Linearity Range (μg / mL)**	**Calibration Equation**	**r2**	**r**
Gallic acid (GA)	0.2-250.0	y = 79,65263x − 21,60843	0.9994	0.9997

Compound - Calibration Curve	Linearity Range (μg / mL)	Calibration Equation	r2	r
Rosmarinic acid (RA)	0.2-250.0	y = 52,88617x – 1,70573	0.9996	0.9998

Table S2. Slope and slope RSD for GA and RA calibration curves.

Compound - Calibration Curve	m	sm	Slope RSD (%)
Gallic acid (GA)	79.653	0.89375	1.122
Rosmarinic acid (RA)	52.887	0.45953	0.8689

Table S3. Intraday and interday precision for gallic acid.

GA Concentration (μg/mL)	Intraday Precision - RSD (%)	Interday Precision – RSD (%)
0.25	4.96	8.41
0.65	4.27	7.19
5	5.71	5.99
120	2.26	6.42
180	2.52	6.16

Table S4. Intraday and interday precision for rosmarinic acid.

RA Concentration (μg/mL)	Intraday Precision - RSD (%)	Interday Precision – RSD (%)
0.25	4.28	7.91
0.65	4.03	6.96
5	5.12	5.91
120	2.06	6.38
180	2.44	6.01

Table S5. Accuracy data for gallic acid.

Concentration (μg/mL)	Obtained Concentration	Accuracy (%)
0.30	0.28	92.6
0.48	0.49	102.9
0.56	0.53	93.8
48	50	104.8
120	117	97.4
206	219	106.1

Table S6. Accuracy data for rosmarinic acid.

Concentration (μg/mL)	Obtained Concentration	Accuracy (%)
0.30	0.28	94.3
0.48	0.49	104.7
0.56	0.52	97.2

Concentration (μg/mL)	Obtained Concentration	Accuracy (%)
48	48	100.5
120	124	103.1
206	216	98.5

Table S7. Robustness data for gallic acid.

Method	Parameter	Parameter Value	Peak Area, mAU	% of Peak Area in Relation to Optimized Method	Retention Time (min)	Difference of Retention Time in Relation to Optimized Method (min)
Optimized variation for robustness evaluation	Formic acid content in water	2.5%	7 037.42	N/A	4.9	N/A
Optimized variation for robustness evaluation	Formic acid content in water	2.8%	6 607.43	93.89	4.9	0.0 min
Optimized variation for robustness evaluation	Flow rate	0.4 mL/min	6 895.56	N/A	4.9	N/A
Optimized variation for robustness evaluation	Flow rate	0.5 mL/min	4 994.45	72.43	3.8	-1.1 min

Table S8. Robustness data for rosmarinic acid.

Method	Parameter	Parameter Value	Peak Area, mAU	% of Peak Area in Relation to Optimized Method	Retention Time (min)	Difference of Retention Time in Relation to Optimized Method (min)
Optimized variation for robustness evaluation	Formic acid content in water	2.5%	4 706.97	N/A	16.9	N/A
Optimized variation for robustness evaluation	Formic acid content in water	2.8%	4 231.56	89.90	16.9	0.0 min

Method	Parameter	Parameter Value	Peak Area, mAU	% of Peak Area in Relation to Optimized Method	Retention Time (min)	Difference of Retention Time in Relation to Optimized Method (min)
Optimized variation for robustness evaluation	Flow rate	0.4 mL/min	4 654.89	N/A	16.9	N/A
Optimized variation for robustness evaluation	Flow rate	0.5 mL/min	3 237.02	69.54	14.0	-2.9 min

Table S9. Stability of gallic acid solutions after 5 and 7 days at −20 °C, 4 °C, and room temperature.

**Condition**	**Concentration (µg/mL)**	**Day 0**	**Day 5**	**Day 5**	**Day 7**	**Day 7**
Condition	Concentration (µg/mL)	Mean (n = 3)	Mean (n = 3)	Stability (%)	Mean (n = 3)	Stability (%)
−20 °C	0.4	30.98	33.64	108.6	33.08	106.8
−20 °C	4	328.48	350.48	106.7	356.07	108.4
−20 °C	120	9 765.20	10 722.18	109.8	9 677.31	99.1
4 °C	0.4	31.68	35.51	112.1	33.92	107.1
4 °C	4	334.44	359.18	107.4	352.49	105.4
4 °C	120	9 724.67	9 656.59	99.3	9 909.43	101.9
Room temperature	0.4	32.66	29.06	88.99	24.13	73.9
Room temperature	4	338.62	351.82	103.9	317.96	93.9
Room temperature	120	9 798.51	10 523.59	107.4	10 425.61	106.4

Table S10. Stability of rosmarinic acid solutions after 5 and 7 days at −20 °C, 4 °C, and room temperature.

**Condition**	**Concentration (µg/mL)**	**Day 0**	**Day 5**	**Day 5**	**Day 7**	**Day 7**
Condition	Concentration (µg/mL)	Mean (n = 3)	Mean (n = 3)	Stability (%)	Mean (n = 3)	Stability (%)
−20 °C	0.4	19.15	20.22	105.6	20.95	109.4
−20 °C	4	215.11	236.0	109.7	229.73	106.8
−20 °C	120	6 263.30	6 889.6	110.0	6 231.98	99.5
4 °C	0.4	22.34	24.98	111.8	24.50	109.7
4 °C	4	209.98	222.78	106.1	220.05	104.8
4 °C	120	6 268.44	6 218.29	99.2	6 400.07	102.1

Condition	Concentration (µg/mL)	Day 0	Day 5	Day 5	Day 7	Day 7
Room temperature	0.4	20.85	18.69	89.65	14.86	71.3
Room temperature	4	210.56	220.03	104.5	198.97	94.5
Room temperature	120	6 256.28	6 775.55	108.3	6 556.58	104.8

S2. Detection Limits and Analytical Performance Summary

Analyte	Range (µg/mL)	R^2	r	LOD (µg/mL)	LOQ (µg/mL)	Accuracy (%)
Gallic acid	0.2–250	0.9994	0.9997	0.0069	0.0212	92.6–106.1
Rosmarinic acid	0.2–250	0.9996	0.9998	0.0105	0.0319	94.3–104.7

S3. LC-MS Evidence Supporting Compound Identification

Table S11. LC-MS characteristics used to support identification of gallic acid and rosmarinic acid.

Peak	Rt (min)	M−H− (m/z)	Compounds	Molecular formula	Fragment	Reference
1	4.832	169	Gallic acid	$C_7H_6O_5$	125, 107, 79	[40, 41, 42]
2	16.786	359	Rosmarinic acid	$C_{18}H_{16}O_8$	197, 179, 161	[40, 41, 42]

Identification was supported by retention-time agreement with authentic standards, extracted-ion chromatograms (EICs), and comparison of observed mass spectra/fragment ions with standards and published literature. The former MRM/SIM wording was removed because it did not reflect the acquisition mode used in this experiment.

S4. Representative Chromatograms

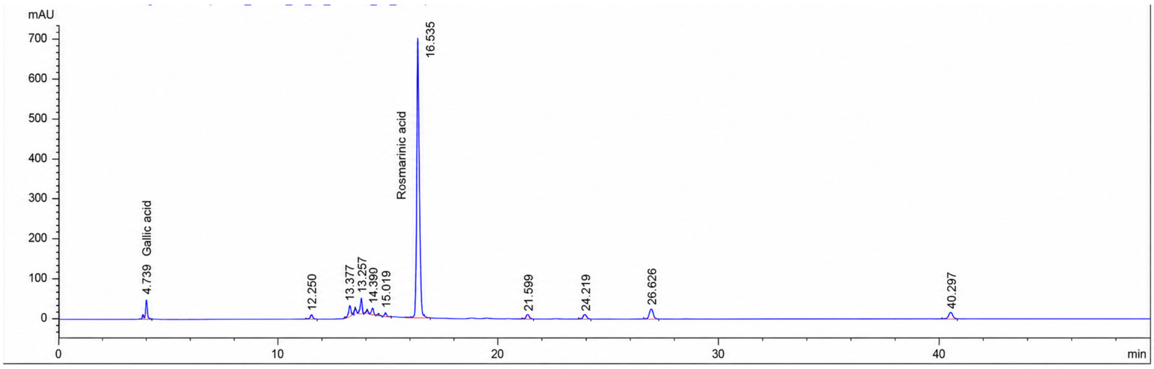

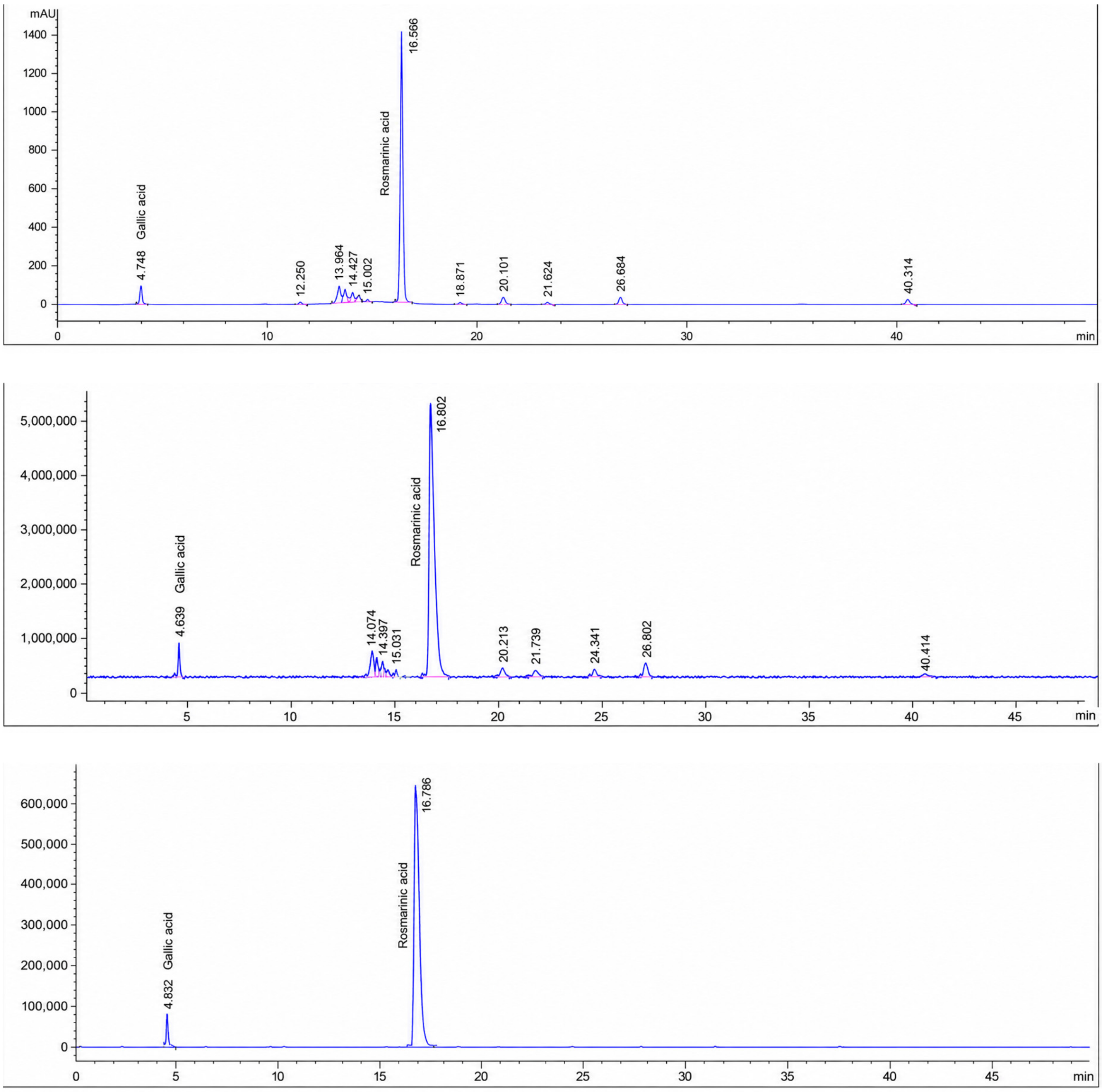

Figure S1. Representative HPLC-UV chromatograms at 360 and 280 nm, total ion chromatogram (TIC), and extracted-ion chromatogram (EIC) used to support identification of phenolic compounds in PV70.

Revision consistency note

Terminology in the revised manuscript has been standardized as follows: Wistar rats (not mice) for the carrageenan experiment; paw mass/paw-mass gain (not paw volume) as the reported endpoint; EIC rather than MRM/SIM for the LC-MS presentation; and ferricyanide reducing-power assay rather than interpreting the reported values as FRAP IC50. These terminology corrections do not alter the underlying numerical results.
